# The effect of stress on prospective memory in robotic command and control

**DOI:** 10.1186/s41235-025-00665-y

**Published:** 2025-08-26

**Authors:** Mollie R. McGuire, Robert S. Gutzwiller

**Affiliations:** 1 Claremont Graduate University, Claremont, CA USA; 2 Information Sciences Department, Naval Postgraduate School, Monterey, CA USA; 3 Arizona State University, Tempe, AZ USA

**Keywords:** Activity-based, Event-based, Prospective memory, Stress, Military

## Abstract

Remembering to carry out an intention at the appropriate time (prospective memory—PM) requires attentional resources that may be limited in stressful circumstances. PM failures in high-risk/high stress environments, such as military operations, can have fatal consequences, and yet, the effect of stress on PM has received little attention. Prior studies that have examined stress and PM used a basic laboratory paradigm that is less applicable to high-risk/high stress environments and have not examined activity-based PM (PM that is elicited by a sequence of events), nor the combined effect of stress and divided attention. The current study examined the effects of stress and divided attention on *event*-based PM (PM that is elicited by environmental cues to signal the appropriate time to perform an intended action) and *activity*-based PM using an applied paradigm with participants remotely piloting robotic reconnaissance missions. Stress and divided attention were manipulated between subjects, and prospective memory was manipulated within subjects. The stress induction was administered prior to the execution of the tasks, with an additional noise stressor continuously on top of the tasks. Divided attention was an auditory odd-digit task during the experimental tasks. *Event*-based PM accuracy was unaffected by stress or divided attention. However, there was an increase in *activity*-based PM accuracy in the high stress condition, while no effect of divided attention was found. These results are the first to demonstrate that stress affects *activity*-based PM, and suggest along with prior literature that stress does not affect *event*-based PM.

## Introduction

Prospective memory (PM), remembering to carry out an intention at the appropriate time, has been a domain of increasing interest in basic memory research (e.g., Einstein & McDaniel, 2005; Marsh & Hicks, 1998; McDaniel & Einstein, 2000). PM failures (failure to complete an intention at the appropriate moment) are prevalent in our everyday lives. Forgetting to follow-up on a business contact, to respond to a student’s e-mail, or realizing you forgot to stop at the grocery store upon arriving home are all examples of PM failures, all of which can be frustrating and inconvenient. While the failures across different environments may be similar, their costs and frequency differ widely. For example, air-traffic controllers operate in an environment where a PM failure has a much higher cost; an air-traffic controller out of LAX forgot to clear one plane for take-off before clearing another to land, causing the planes to collide and leaving 34 people dead (Dismukes & Nowinski, 2007).

Unique environments can create conditions of frequent and high-cost PM failures. Pilots, air-traffic controllers, emergency room personnel, and military operators all work in environments where these memory failures can lead to fatalities, in part because of information overload due to multitasking, and stress (Dismukes, 2008; Grundgeiger et al., 2014; Loft et al., 2013). Therefore, it is important to understand the effects of stress on prospective memory with the applicable population. Most research examines PM using memory research paradigms in a basic laboratory setting using tasks with limited ecological validity, and undergraduate students as participants. In the typical laboratory paradigm created by Einstein and McDaniel (1990), participants (a) receive instructions and practice for an ongoing task, (b) receive PM instruction, (c) then perform an intermediate task, (d) are reminded of the ongoing task, and (e) perform the ongoing task with an embedded PM task. There are several adaptations from the basic paradigm, but all follow a similar structure (e.g., Einstein et al., 2005; Knight et al., 2011; Smith, 2003). The present study used this structure but created an applied variant by using simulated military operations to test the effects of stress and divided attention on PM, using primarily military officers as participants.

### Multiprocess theory of prospective memory

According to the *multiprocess theory*, successful PM can be supported by both monitoring and spontaneous retrieval processes, those that are not top-down initiated, but more reactive to a stimulus (McDaniel & Einstein, 2000). Whereas monitoring processes guide the scan of the environment for cues, spontaneous retrieval processes react to cues in the environment; these environmental cues trigger the retrieval of the intention (e.g., seeing a grocery store on the way home “cues” the intention to buy groceries). The type of PM and the context influence whether monitoring or spontaneous retrieval processes are relied on to support PM (Einstein et al., 2005). Both processes require attentional resources, but because monitoring requires an intention to be kept in mind, it requires more than spontaneous retrieval processes (McDaniel & Einstein, 2000). Even though spontaneous retrieval processes are signaled by an external cue, they do still require some amount of attentional resources. For example, Harrison et al. (2014) encouraged participants to rely on spontaneous retrieval processes through instruction manipulations, and then examined whether a divided attention task would disrupt PM. In Experiment 2 of their study, spontaneous retrieval processes were disrupted by a highly demanding divided attention task (i.e., random number generation task). However, in Experiment 1, a moderately demanding divided attention task failed to interfere with PM, suggesting that spontaneous retrieval processes require some amount of attentional resources, but the demand level is critical in invoking interference and must be high.

When a divided attention task competes for the same attentional resources as a PM task, a PM failure may occur due to attentional demands that exceed capacity (Marsh & Hicks, 1998). A similar observation is made in studies where participants are keeping an intention in mind (i.e., monitoring; Einstein et al., 2003) and for those relying on spontaneous retrieval processes (Harrison et al., 2014).

### Prospective memory tasks

Similarly, different types of PM tasks (*event*-based, *activity*-based, and *time*-based) may differ in the amount of attentional resources required to be successfully carried out (Kvavilashvili & Ellis, 1996; see also Brewer et al., 2011, McDaniel & Einstein, 2007). *Event-*based PM relies on environmental cues to signal the appropriate time to perform an intended action (e.g., seeing a co-worker in the hallway may remind a person of information they intended to pass along to them). *Activity-*based PM can rely on sequentially oriented task contexts, where the intention is related to performing a task before or after another task (e.g., a person may have the intention to go to the post-office after they eat lunch, but before returning to work). Finally, *Time-*based PM tasks can rely on intention triggered by a certain time, or in a certain amount of time (e.g., a person may have the intention to go to a meeting at 1600, or call their spouse in 5 min).

Types of PM tasks differ in the effort imposed on the individual; for example, in *activity*- and *time*-based tasks, self-initiation is required to monitor (a) one’s own actions in *activity*-based, or (b) the time or passage of time in *time*-based. Because of this, *time*- and *activity*-based PM tasks require more attentional resources than *event*-based PM tasks (Brewer et al., 2011; Kliegel et al., 2005; Nater et al., 2006). Because the attentional resources needed differ depending on the type of PM task, and stress depletes attentional resources, stress may differentially affect PM depending on the type of task.

### Stress effects

Stress effects on cognitive resources do not always mirror the results of human performance. For example, the physiological response to stress results in a decline in attentional resources (Arnsten, 1998). Yet performance on attention-related tasks has been shown to increase under stress (Booth & Sharma, 2009; Chajut & Algom, 2003), perhaps due to more effective resource allocation (Chajut & Algom, 2003) similar to the strategy of “task shedding” observed in high workload situations (Goteman & Dekker, 2006; Grier, 2008). Some high stress situations reduce visual scanning behaviors and lead to missing hazards in working environments (Pooladvand & Hasanzadeh, 2023). In a study on perceived stress and state anxiety, Petrac et al. (2009) found that higher perceived stress decreased divided attention performance in an auditory task, but that higher state anxiety resulted in an increase in performance. Combined these studies suggest that the type of stress as well as the type of task are important in understanding how stress might affect attentional focus.

The effect of stress on memory is nuanced as well. In a meta-analysis by Shields et al. (2017), they found that episodic memory was differentially affected by stress depending on when the stressor was encountered (i.e., prior to encoding or post encoding) as well as the timing and relevance of the stressor to the task. This is important to prospective memory as the encoding phase or phase where you set your intention might be deleteriously affected by stress if the stressor is encountered prior to encoding, or it could improve memory if the stressor is encountered after the intention was set. On the retrieval end, according to Shields et al., retrieval is negatively impacted by stress. In understanding how stress affects prospective memory, it is clear that it might be multifaceted, and the placement of the stressor is critical to the observed outcome. Additionally, the type of stressor might be a factor in how severe the effects are. In a review on stress and memory, Sandi (2013) reported that when intrinsic stress (task-related) and extrinsic stress (task-unrelated) effects are combined, negative effects on memory are more severe than intrinsic stress alone.

The effect of acute stress on PM has been investigated in a handful of laboratory experiments, but the results are a bit mixed indicating more research is needed. In the existing work, it remains important to distinguish effects by PM task type (because they determine demands), and by the stress induced. For example, Nater et al. (2006) tested *time*- and *event*-based PM in stress versus no-stress conditions, using the Trier Social Stress Test (TSST). *Time*-based PM was better during the stress than the rest condition, but there was no difference in *event*-based PM between the stress conditions. As suggested above, this difference may be due to more resources needed to support *time*- than *event*-based PM. In a study by Walser et al. (2013) also using TSST (and a no-stress TSST matched scenario) found no difference in *event*-based PM performance between stress conditions. However, Szőllősi et al. (2017) found that stress increased reaction times in event-based prospective memory, but found no difference in time-based prospective memory, suggesting that automatic processes were affected by stress, but not executive processes. While Möschl et al. (2017) found that stress did not affect PM performance, they also looked at commission errors and monitoring costs. They found that when task demand was high that stress led to a reduction in monitoring costs but an increase in commission errors. They suggest that stress prompts a shift toward automatic processing that increases response time but comes with a cost in commission errors in the absence of a PM intention.

The previous studies on stress and PM all had a stressor prior to the testing. An important difference in a real-world stress situation is that cognitive tasks often have to be performed *simultaneously* with the stressful event. It is unclear how a constant stressor might add to the stress dimension.

Finally, prior studies have only examined *event*- and *time*-based PM under stress. *Activity*-based PM has not yet been examined; if different types of PM tasks require different amounts of attentional resources, and if the effect of stress may be more detrimental to one than the other, it is important to characterize *activity*-based PM as well.

Because multitasking is commonplace, and necessary in many high-risk and high stress conditions, we also included a divided attention task. If stress consumes attentional resources, the effect of a divided attention task should be to lower PM accuracy in the stressed group (e.g., Harrison et al., 2014, though we used a slightly easier task).

The current study examined how stress and divided attention affect prospective memory accuracy for two different types of prospective memory tasks (*event*-based and *activity*-based) using a military task, in which participants remotely operated robots through a reconnaissance mission. Navigation of a real-world robot via the robot’s video feed was chosen for the current study because (a) it allowed participants to engage in a real task (versus a virtual simulation), (b) the ongoing and PM tasks could still be adapted from prior PM studies, and (c) the navigation task is representative of real-world military tasks, but simple enough to allow for controlled laboratory testing conditions and require only a small amount of training.

## Methods

### Participants and design

The design was a 2 (stress: high, low) × 2 (attention: divided, non-divided) × 2 (block: PM, control) mixed factorial design with stress and attention manipulated between subjects, and block manipulated within subjects. Based on this design, a power analysis by G*Power (Faul et al., 2007) determined 152 participants were needed with an effect size, ${\eta }_{p}^{2}$= 0.07 (effect size based on a significant main effect of divided attention on prospective memory accuracy; see Harrison et al., 2014, additional analysis after Experiment 3), statistical power = .80, and alpha = .05. A total of 117 participants from the Naval Postgraduate School participated in the study and received no compensation. However, only 101 participants were included in the analysis (*M*_*age*_ = 34; 85 males, 16 females; 88 active duty officers). Two participants were excluded because of failed manipulation checks on the *event*-based PM task, 13 participants were excluded because of failed manipulation checks on the *activity*-based PM task, and one participant was excluded due to technical issues with the auditory task.

### Ongoing tasks

PM studies often simulate real-life contexts where PM cues occur while people are engaged in an activity (an ongoing task). Participants conducted two ongoing tasks sequentially in the experiment, a categorization task and a robot navigation task.

#### Robot navigation task

**Fig. 1:**
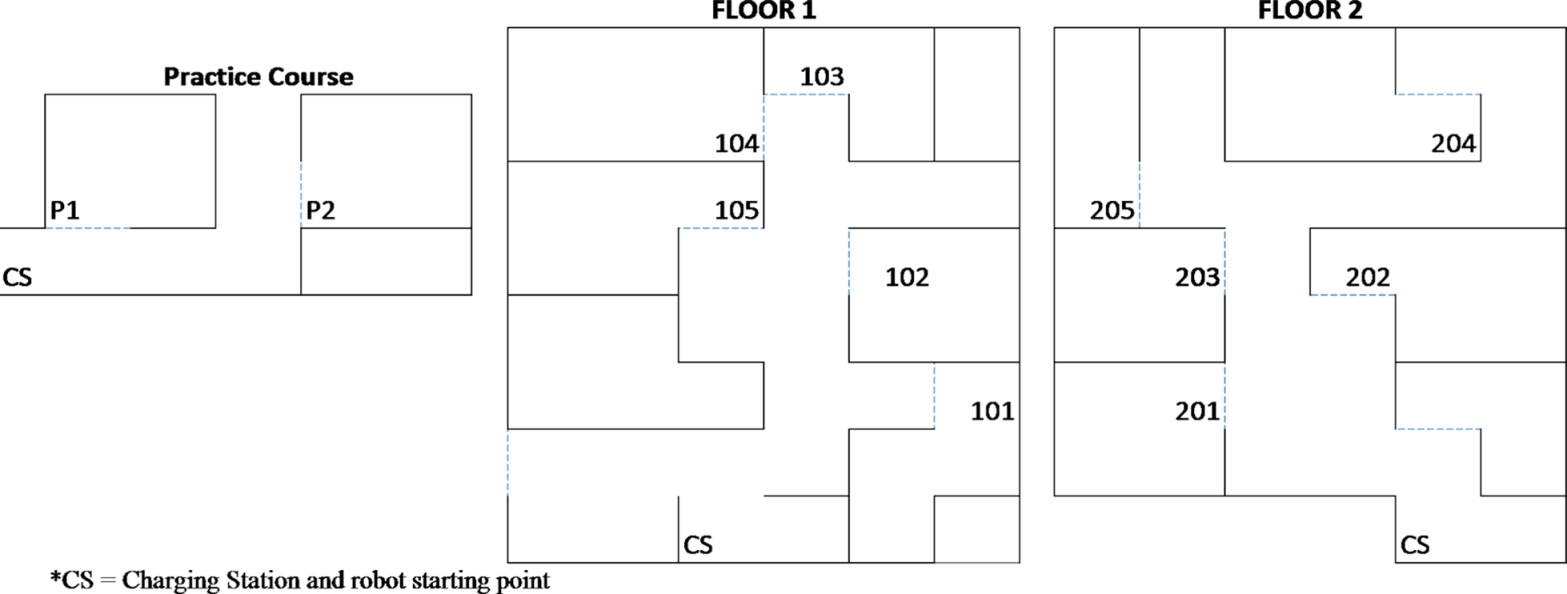
Layout of the navigation courses. Participants navigated the practice course during the practice phase. Floor 1 and Floor 2 were counterbalanced. Numbers represent room numbers in the order participants navigated through the floor

There were three different robot courses that participants navigated in the experiment; (a) the practice course, (b) “floor one,” and (c) “floor two.” The practice course had two rooms, and each “floor” had five rooms (see Fig. 1). Participants navigated through one “floor” per block, half starting with “floor one” and the other with “floor two.”

In the practice course, participants first practiced robot navigation, and in each room, were told to locate an inner door. After practicing navigation alone, participants re-navigated the robot through the practice course, now incorporating the categorization task (and divided attention task for those assigned to the divided attention condition).

Navigation was completed using the real-time video feed from the robot and an outline of the “floor” with room numbers for reference. Participants had a separate monitor for the navigation feed and controlled the robot using the arrows on the keyboard.

#### Categorization task

Participants categorized identification badges^1^ as either military or civilian by pressing “mil” or “civ” on a keyboard (labels placed on the “g” and “f” keys, respectively). Categorization of military or civilian was based on the rank or credentials on the badge; military had a rank below their name (e.g., LT and CPT), and civilian had either credentials (e.g., PhD, RN, and MD) or the area was blank. ID badges were presented one at a time,^2^ and participants identified 30 badges per room. The ID badges remained on the screen until a response was made, with each preceded by a brief 500 ms fixation cross, and a 500 ms inter-stimulus interval between badges.

### Prospective memory tasks

The categorization task was used as one ongoing task in which the *event*-based PM task was embedded. The *activity*-based PM task was to be completed during transition between finishing the categorization task and resuming the navigation task.

#### *Event*-based prospective memory task

The *event-*based PM task was a modified version of the PM tasks commonly used, where a PM target was embedded in an ongoing task and signals that an alternate action should be taken (e.g., Harrison et al., 2014; Hicks et al., 2005). The *event-*based PM task required participants to press the “q” key when they encountered a medical doctor (as indicated by “M.D.”) during the categorization task instead of the military/civilian determination. Four *event*-based PM targets were presented, the first occurring randomly in the second room, and all following targets were presented in each subsequent room, no less than 30 badge presentations apart from prior targets.

#### *Activity*-based prospective memory task

In the *activity-*based PM task, participants were required to state the room number that they were in after they finished the categorization task, but before they navigated to the next room, for a total of five *activity*-based PM responses. In other words, the activity-based PM task took place in between the two ongoing tasks.

### Divided attention task

Participants were also randomly assigned to either the divided or non-divided attention condition. Participants in the divided attention condition heard single digits spoken at a rate of 2 s apart and indicated on a separate interface tablet device when they heard two odd digits in a row (odd-digit task; Harrison et al., 2014). The odd-digit task was performed concurrently with the navigation and categorization tasks.

### Intermediate tasks

Intermediate tasks were given after PM instructions, but before the start of the categorization task so that the PM tasks did not become vigilance tasks. The intermediate tasks also served as an opportunity to begin inducing stress in the high stress group. There were two versions of the intermediate task, counterbalanced so that the participant completed one before Block 1 and the other was completed before Block 2. The intermediate tasks consisted of 20 problems, either (a) 10 series completion and 10 odd word out problems, or (b) 10 arithmetic correct/incorrect and 10 analogy problems.

### Stress manipulations

Participants were randomly assigned to the high or low stress condition. To elicit stress, a combination of stress methods was used in the intermediate task period: (a) time pressure, (b) difficulty, and (c) inducing threat to ego, which together have been used together to manipulate stress in the previous studies (Chajut & Algom, 2003; Keinan et al., 1999; Steinhauser et al., 2007). These tasks were chosen as an alternative to the more common method (i.e., TSST), because the participant population (military officers) had experience with public speaking, and informal interviews confirmed that it was unlikely to induce stress.

Participants in the *high stress* condition were told that the problems were a measure of cognitive ability and would be compared with their peers at the end of the experiment (threat to ego). They were given a “unique” three-digit ID that they were told was their “personal number associated with their responses,” (personal relevance) and given difficult, and sometimes impossible to solve questions (difficulty). In contrast, participants in the *low stress* condition were told the task helped assess the psychometric properties of the test, and their individual performance is of no interest. They were also told all answers were anonymous and given easy to moderate questions. Participants in both conditions were given a 30 s time limit per question, this was ample time for the lower stress condition, but induced time pressure in the higher stress condition.

#### Continuous stressor

Noise has been shown to induce stress (Booth & Sharma, 2009; Chajut & Algom, 2003), especially uncontrollable noise (Banis & Lorist, 2012; Henderson et al, 2012). Participants in the *high stress* condition heard uncontrollable, continuous white noise at approximately 70–80 dB via headphones throughout the experiment. Participants in the *low stre*ss condition were not exposed to the white noise.

### Stress measures

Several methods of stress measurement were used to quantify the stress manipulation. Participants completed the *state* section of the State-Trait Anxiety Inventory (STAI-S; Spielberger et al., 1970) as the subjective measure of anxiety. Participants completed the STAI-S autonomously on a computer, before and after the experiment. Additional subjective measures were taken at the end of the exit questionnaire; participants rated on a 1–7 Likert scale, (a) how stressful the experiment was in general, (b) how stressful the intermediate tasks were, (c) how stressful the constant white noise was, and (d) how difficult the intermediate tasks were. Heart rate variability (HRV) was continuously recorded and used to monitor changes in stress states (e.g., Hernandez et al., 2014). For all indices used to measure HRV, see McGuire (2016).

### Procedure

**Fig. 2:**
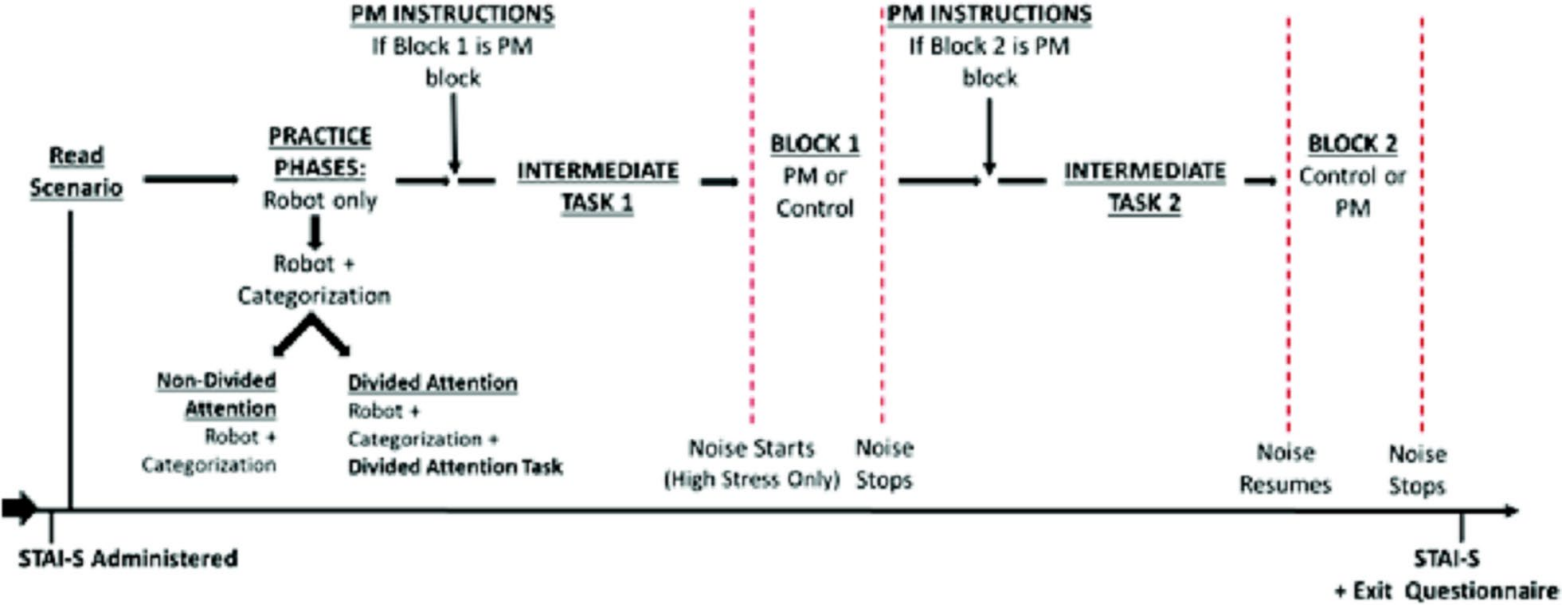
General experimental procedure. PM = Prospective memory. Dotted vertical lines bound when the high stress condition additionally experienced noise

During the experiment, participants navigated a robot through a series of rooms. Once inside the rooms, participants switched from robot navigation to a categorization task. During the PM block, participants also had to perform *event*-based and *activity*-based PM tasks. Participants in the divided attention condition also had to complete a divided attention task with the ongoing tasks in both the control and PM block. Stress and divided attention were varied between subjects, creating four conditions (high stress/divided attention, high stress/non-divided attention, low stress/divided attention, or low stress/non-divided attention). Stress was induced by using a combination of (a) psychosocial stressor, administered before the PM and control blocks, and (b) a noise stressor administered during the blocks. The general procedure is outlined below (see Fig. 2).

After providing consent, participants received a wristband that monitored HRV. They then completed the STAI-S measure. After assignment to their condition, participants were read aloud their “mission” (scenario) that detailed tasks they would carry out during the experiment and then completed robot navigation practice to familiarize themselves with navigational controls and course esthetics. Participants then received specific instructions for the categorization task. Participants then navigated back through the practice course, integrating the categorization task, and the divided attention task in the divided attention condition. Participants were read aloud the PM task instructions immediately before the intermediate task preceding the PM block, either Block 1 or Block 2 depending on their randomly assigned condition. After the first block, participants completed another intermediate task before beginning the second block. The STAI-S was then re-administered, and the HRV wristband was removed. Participants then completed a recall test and exit questionnaire and were debriefed.

#### Recall test and exit questionnaire

Following the second STAI-S administration, participants completed a recall test and then an exit questionnaire. The recall test was a manipulation check to ensure that failures to complete the PM tasks were due to prospective and not retrospective memory failures (Loft et al., 2013; Smith, 2003). Participants who did not execute the PM tasks and indicated that they did not remember the PM instruction were removed from analyses. After the recall test, participants were asked how much importance they assigned to each task, and whether they had been through any military stress training. Other manipulation check questions were (a) how engaged they were during the experiment, (b) whether they had any problems hearing sounds from the headphones, (c) if they had any hearing problems, (d) if they owned or had experience navigating a ground robot, and (e) if they regularly played first-person shooter games.

### Measurements

Event-based PM accuracy. Event-based PM accuracy was calculated as the proportion correct out of four possible responses (counted as correct if they were made in response to the target, or on the immediate categorization trial after the target).

#### Activity-based PM accuracy

Activity-based PM accuracy was calculated as the proportion correct was out of five possible responses (counted as correct if responses were made after the classification task was complete, but before the participant left the room).

#### Ongoing task reaction times

To assess whether participants were relying on monitoring or spontaneous retrieval processes for successful PM, ongoing task reaction times were compared between the PM and control block. Whereas slower ongoing task reaction times in the PM than control block is indicative of a reliance on more monitoring than spontaneous retrieval processes, no difference in reaction times is indicative of a reliance on more spontaneous retrieval. Methods were used to try to encourage a reliance on spontaneous retrieval processes (see McGuire, 2016, for rationale and methods).

Distribution of the ongoing task reaction times were found to be positively skewed and therefore transformed via log transformations for analyses. Descriptive statistics are reported untransformed. Two univariate outliers were identified using the IQR approach both before and after transformation, and therefore removed, and no multivariate outliers were identified.

#### Inter-beat interval (RR)

RR is one measure of HRV and was assessed to determine the stress reaction to (a) the intermediate task (pre-task stressor) and (b) the white noise (ongoing stressor). To assess the intermediate task stressor, measures from before the stress manipulation (practice) were compared to measures during the stress manipulation (Intermediate Task 1). To assess the white noise stressor, measures from the practice phase were compared to measures from Block1.

#### STAI-S

The STAI-S is a 1–4 scale and was scored per the instructions on the scoring sheet (Spielberger et al., 1970).

#### Self-reported stress

The self-reported stress scale on stress and difficulty of the experiment was assessed on a 7-point Likert scale.

## Results

### Manipulation checks

Both physiological and subjective measures were assessed to determine whether stress or anxiety was successfully manipulated.

#### Physiological measures

RR was used to assess (a) the pre-task stressor and (b) the white noise stressor. Both analyses were a 2 (Stress: high, low) × 2 (Attention: divided, non-divided) × 2 (Time of measurement: practice phase, stressor) repeated measures ANOVA. In the comparison between the practice phase and the intermediate task 1, there was a main effect of time of measurement, RR was lower after the stressor (*M* = 867.55, *SD* = 148.92, CI_95_[836.32, 898.79]) than before the stressor (*M* = 891.75, *SD* = 160.41, CI_95_[858.08, 925.41]), *F* (1,88) = 13.77, *p* < 0.001, ${\eta }_{p}^{2}$=0.135. There was an interaction with time of measurement (Practice phase vs Intermediate task 1) and stress, *F*(1, 88) = 5.95, *p* = 0.017, ${\eta }_{p}^{2}$=0.063. Whereas RR in the high stress condition was significantly lower during Intermediate Task 1 (*M* = 857.53, *SD* = 133.97, CI_95_[813.38, 901.67]) compared to the Practice phase (*M* = 895.55, *SD* = 145.06, CI_95_[847.96, 943.14]), *t*(45) = 4.18, *p* < 0.001, *d* = 0.62), there was no difference between Intermediate and Practice phases in the low stress condition, *t*(45) = 0.99, *p* = 0.327. Therefore, participants displayed changes in RR associated with increased stress in the high, but not the low stress group. While the same pattern was found when assessing the white noise stressor, there was also an interaction between time of measurement and attention, *F*(1, 90) = 8.09, *p* = 0.006, ${\eta }_{p}^{2}$=0.082. RR was lower in Block 1 than the Practice phase for both attention conditions. However, in the divided attention condition, there was a larger decrease in RR, *p* < 0.001, d = 0.78 than the non-divided attention condition, *p* = 0.038, d = 0.32, indicating that the white noise increased participants stress overall, but more so in the divided attention condition, which may indicate that the divided attention task added to the overall stress of the participant. See McGuire, 2016, doctoral dissertation for additional physiological results.

#### STAI-S

A 2 (Stress: high vs low) × 2 (Attention: divided vs non-divided) × 2 (Time of measurement: beginning vs end of experiment) repeated measures ANOVA was conducted on the STAI-S scores. Scores were higher on the post-experiment STAI-S (*M* = 31.58, *SD* = 7.76, CI_95_[30.07, 33.04]), than pre-experiment (*M* = 27.09, *SD* = 5.56, CI_95_[25.98, 28.17]), *F*(1, 97) = 55.23, *p* < 0.001, ${\eta }_{p}^{2}$= .36, suggesting a general increase in subjective anxiety. Additionally, scores were higher on the STAI-S in the divided attention (*M* = 30.70, *SD* = 6.65, CI_95_[29.07, 32.33]), than the non-divided attention condition (*M* = 27.93, *SD* = 6.35, CI_95_[26.29, 29.58]), *F*(1, 97) = 8.74, *p* = 0.004, ${\eta }_{p}^{2}$=0.083, suggesting the divided attention condition increased subjective anxiety more than the non-divided attention condition, regardless of stress condition. The main effect of stress condition was not significant, *F* < 1, *p* = 0.546, ${\eta }_{p}^{2}$=0.004, nor was the interaction of stress and attention, *F*(1, 97) = 1, *p* = 0.319, ${\eta }_{p}^{2}$=0.01. This supports the idea that there was a general increase in subjective anxiety in the experiment, with divided attention increasing more than undivided conditions, but no difference in subjective anxiety between stress conditions.

#### Self-report stress measures

Finally, self-report measures were assessed with a MANOVA. There were significant main effects of stress, *F*(1, 95) = 25.72, *p* < 0.001, ${\eta }_{p}^{2}$=0.448, and divided attention, *F*(1, 95) = 8.98, *p* < 0.001, ${\eta }_{p}^{2}$=0.221. Follow-up univariate ANOVAs were performed on each self-report measure. In the general stress self-report measure, a main effect of attention was found; higher general stress was reported in the divided (*M* = 3.67, *SD* = 1.38, CI_95_[3.32, 4.00]) than non-divided attention condition (*M* = 2.47, *SD* = 1.03, CI_95_[2.13, 2.82]), *F*(1, 97) = 23.84, *p* < 0.001, ${\eta }_{p}^{2}$=0.197. In the problem stress self-report measure, a main effect of stress was found; higher stress was reported in the high (*M* = 3.96, *SD* = 1.41, CI_95_[3.59, 4.33]) than the low stress condition (*M* = 2.75, *SD* = 1.25, CI_95_[2.38, 3.12]), *F*(1, 97) = 21.12, *p* < 0.001, ${\eta }_{p}^{2}$=0.179. In the problem difficulty self-report measure, there was a main effect of stress; higher difficulty was reported in the high (*M* = 5.27, *SD* = 1.31, CI_95_[4.90, 5.64]) than the low stress condition (*M* = 3.12, *SD* = 1.32, CI_95_[2.75, 3.48]), *F*(1, 97) = 68.33, *p* < .001, ${\eta }_{p}^{2}$=0.413. Subjective measures of problem stress and problem difficulty confirmed that the intermediate task questions were subjectively (a) more stressful and (b) more difficult in the high compared to the low stress condition.

### Prospective memory accuracy

Means and standard deviations for all PM accuracy are presented in Table 1.

**Table 1:** Mean responses for prospective memory accuracy

	Event-Based		Activity-Based	
	Non-DA	DA	Non-DA	DA
	Mean	Mean	Mean	Mean
Low Stress	0.96 (0.12)	0.89 (0.19)	0.50 (0.45)	0.42 (0.41)
	[0.90, 1.02]	[0.82, 0.95]	[0.34, 0.67]	[0.26, 0.59]
High Stress	0.95 (0.13)	0.91 (0.18)	0.68 (0.45)	0.79 (0.39)
	[0.89, 1.01]	[0.85, 0.97]	[0.51, 0.85]	[0.62, 0.96]

Standard deviations are in parentheses; 95% confidence intervals are in square brackets. DA = Divided attention

#### Event-based PM

A 2 (Stress: high vs low) × 2 (Attention: divided vs non-divided) ANOVA was run on hit rates. *Event*-based PM accuracy did not significantly differ in the divided (*M* = .90, *SD* = .18) than non-divided attention condition (*M* = .96, *SD* = .12), *F*(1, 97) = 3.47, *p* = .066, ${\eta }_{p}^{2}$= .035. There was also no effect of stress, F < 1, *p* = .80, ${\eta }_{p}^{2}$= .001; and no interaction between stress and attention F < 1, *p* = .569, ${\eta }_{p}^{2}$= .003.

#### Activity-based PM

As before, a 2 (Stress: high vs low) × 2 (Attention; divided vs non-divided) ANOVA was run on hit rates. Whereas no-stress effects were found for *event-*based PM, there was a main effect of stress in *activity-*based PM accuracy, but in the opposite direction from what was predicted; *activity*-based PM was higher in the high (*M* = .74, *SD* = .42, CI_95_[.62, .86]) than low stress condition (*M* = .46, *SD* = .42, CI_95_[.35, .58]), *F*(1, 97) = 10.47, *p* = .002, ${\eta }_{p}^{2}$= .097. This result indicated higher stress increased *activity-*based PM accuracy. There was no effect of divided attention *F* < 1, *p* = .85, ${\eta }_{p}^{2}$= .00, nor interaction between stress and attention conditions *F*(1, 97) = 1.31, *p* = .26*,* ${\eta }_{p}^{2}$= .013.

### Ongoing task performance

A 2 (Stress: high vs low) × 2 (Attention: divided vs non-divided) × 2 (Block: PM vs control) ANOVA was conducted (means and standard deviations are presented in Table 2). Reaction times were faster in the control than the PM block, *F*(1, 95) = 74.98, *p* < 0.001, ${\eta }_{p}^{2}$=0.44, suggesting that participants were relying more on monitoring in the PM condition than in the control condition. Additionally, reaction times were significantly slower in the divided than non-divided attention condition, *F*(1, 95) = 64.46, *p* < 0.001, ${\eta }_{p}^{2}$=0.40. This is consistent with the previous research on ongoing task reaction times in divided versus non-divided attention (Harrison et al., 2014). Moreover, there was a small but significant interaction between block and stress, *F*(1, 95) = 4.35, *p* = 0.04, ${\eta }_{p}^{2}$=0.04. However, follow-up tests failed to find significant differences between stress conditions within each block (PM block, *t*(97) = .82, *p* = 0.41; control block, *t*(97) = 0.03, *p* = 0.97). Finally, there was no interaction between block and attention, *F*(1, 95) = 3.86, *p* = 0.053, ${\eta }_{p}^{2}$=0.04, nor was there a three-way interaction between block, stress, and attention, *F*(1, 95) = 3.31, *p* = 0.072, ${\eta }_{p}^{2}$=0.03.

**Table 2:** Ongoing task reaction times (in ms)

	Control block		Prospective memory block	
	Non-DA	DA	Non-DA	DA
	Mean	Mean	Mean	Mean
Low Stress	721.28 (126.57) [633.28, 809.27]	1037.61 (265.70) [949.17, 1125.60]	845.65 (184.74) [732.67, 958.62]	1131.66 (403.30) [1018.69, 1244.64]
High Stress	720.90 (214.29) [631.09, 810.71]	1038.51 (252.57) [950.51, 1126.50]	765.32 (168.16) [650.01, 880.62]	1117.62 (310.80) [1004.64, 1230.60]

Standard deviations are in parentheses; 95% confidence intervals are in square brackets. DA = Divided attention

### Exploratory analysis

A plausible confound that has been proposed is that divided attention might be acting as an additional stressor. In order to address this concern, four groups were created for a one-way ANOVA. The groups were: (a) high stress/DA, (b) high stress/noDA, (c) low stress/DA, and (d) low stress/noDA. There was a difference among the groups for activity-based prospective memory, *F*(3, 100) = 3.690, *p* = 0.010. There were no differences between DA in either the low or the high stress conditions, *p* = 0.250 and *p* = 0.176, respectively. The high stress conditions were both significantly higher than the low stress/divided attention condition in activity-based prospective memory accuracy, *p* = 0.019 (high stress/noDA) and *p* < 0.001 (high stress/DA). There was no difference on *event*-based prospective memory, *F*(3, 100) = 1.297, *p* = 0.28.

Further, an exploratory analysis between potential groups based on the exit questionnaire data revealed three that are notable. First, the effect of stress on activity-based PM was examined between those with and without military training. The effect of stress on activity-based PM held for those that had been through military training (n = 61, *stress condition*: *M* = 0.74, *SD* = 0.43; *non-stress condition*, *M* = 46, *SD* = 44), *F*(1,57) = 6.79, *p* = 0.012, ${\eta }_{p}^{2}$= .11, but not for those that had not been through stress training (n = 40, *stress condition*, *M* = 0.72, *SD* = 43; *non-stress condition*, *M* = 47, *SD* = 41), *p* = 0.097. Second, while the effect of stress on activity-based PM held for those that do not regularly play first-person shooter games (n = 63, stress condition, M = 0.82, SD = 0.34; *non-stress condition*, *M* = 0.44, *SD* = 0.41), *p* < 0.001, the effect of stress on activity-based PM was not found in those participants who identified as regularly playing first-person shooter games (n = 38, *stress condition*, *M* = 0.62, *SD* = 0.50; *non-stress condition*, *M* = 0.52, *SD* = 0.45), *p* = 0.48. Finally, when looking at whether the prospective memory block was the participants first or second block, the activity-based PM effect was only found when it was their second block, *p* < 0.001. Cortisol takes 20–30 min to peak after stressor onset, and this effect is consistent with the timing of the release of cortisol after stressor onset.

## Discussion

Prospective memory (PM) in high stress environments, such as military operations, is an important area of research because the consequences of a PM failure can be fatal. Yet this topic has received little attention in empirical research, and the results of the limited research have shown no effect of stress on *event*-based PM (Nater et al., 2006; Walser et al., 2013). Stress and PM are both multifaceted phenomena. As a result, relationships between them require more research to fully understand. The study reported here explored the relationship between PM and stress, finding that *activity*-based PM accuracy increases under stress. Our results further suggested that *event*-based PM tasks requiring little attentional resources may be more resistant to the effects of stress. However, these effects should be interpreted in light of the subjective reporting of the participants, which suggested that the stress manipulation was not as extreme as some in the real world. For example, the high stress group reported stress caused by the intermediate task as being low-moderate, stress caused by the white noise being low, and the general problem difficulty as moderate. Therefore, it seems the stress in this experiment could be more reflective of moderate rather than high stress. The moderate stress reported could also be due to the subject population, which consisted of mid-level military officers, including pilots, and “boots-on-the-ground” leaders with one or more combat tours. The laboratory-style stressors may not have been sufficient to induce a higher level of stress. Examining PM in a naturally high stress environment (e.g., military survival and evasion training) could be used to examine whether PM under high levels of stress results in a decline in PM performance, showing the same inverted U-shaped distribution observed in retrospective memory.

### *Activity*-based prospective memory accuracy

To our knowledge, ours is the first study to examine stress and *activity*-based PM. Of particular interest is that we found *activity*-based PM accuracy was higher in the high stress condition, opposite our predictions. However, the effect of stress was consistent with the results of Nater et al. (2006), showing an increase in *time*-based PM performance in a stress versus a non-stress condition. Also both *activity*- and *time*-based PM require more attentional resources than *event*-based PM, as they do not have an environmental cue, and require more self-initiation to monitor one’s own actions or the time (Brewer et al., 2011; Khan et al., 2008; Nater et al., 2006). The increase in accuracy under stress seen both in *activity*-based PM in the current study, and in the *time*-based task in Nater et al., suggests that the more resource-demanding PM tasks might benefit from the focused narrowing of attention induced by stress. This could lead to a focus of attentional resources toward PM monitoring, even under divided attention conditions.

### *Event*-based prospective memory accuracy

There was no effect of stress or divided attention on *event*-based PM accuracy; though the main effect of attention was approaching significance, such that accuracy was lower in the divided attention condition. In general, the pattern fits with the previous research indicating that divided attention disrupts monitoring processes. *Event*-based PM requires varying levels of attentional resources depending on a multitude of variables, such as relatedness of the environmental cue and the intention, importance, and associativity (McDaniel & Einstein, 2000). Theoretically, the *event*-based PM task in this experiment required very little attentional resources (i.e., there was only one target, the target was distinctive, and the PM task was highly associated with the ongoing task). This may be one explanation for why no effect of stress was seen with this task, and why the overall accuracy was nearing ceiling (*M* = 0.93). A more attentionally demanding *event*-based PM task (e.g., multiple targets, less distinctive target, and less associated with the ongoing task) is needed to examine whether *event*-based PM would increase under a moderate amount of stress, showing the same pattern as observed with *activity*-based PM.

### Military relevance

This study employed a remotely operated robotic reconnaissance mission as an experimental paradigm to increase the ecological validity by mirroring a realistic military task. Operating a robot within the context of military operations currently requires multitasking in extreme stress environments. Using an experimental design that mirrors the types of tasks used in these environments is necessary to examine the nature of potential PM failures. Stress experienced in these environments was thought to have a deleterious effect on PM on the outset of the study. However, it was found that stress may in fact elicit a more resource focused attention strategy in military officers. The effect was particularly seen in those that had been through military stress training. This argues for the effectiveness of such trainings in preparation for remembering intentions in combat environments. However, given that this was a subset of the total sample in this study, future research should look into those with military stress training, at various lengths of time post-stress training to see if there is a difference compared to those with no-stress training, and how the difference might vary with time and experience.

### Limitations and future directions

The effect of stress on activity-based PM was found in those who had been given the PM task in the second block. The timing of when the participants received the stressor to the time they were engaged in the second block is quite consistent with the research on the effect of cortisol on retrieval. Retrieval has been shown to be disrupted by the release of cortisol at approximately 25 min after stressor onset (Schwabe & Wolf, 2014). The participants who received the PM tasks in the first block were performing the tasks too close to the stressor onset to have been affected by the release of cortisol that would have started at stressor onset. Future research should nevertheless take this timing into account when examining the effect of stress on prospective memory. Additionally, the noise stressor acted as an additional stressor that may have differentially affected performance with divided attention. Petrac et al. (2009), for example, found that divided attention was disrupted during an auditory task by perceived stress. The continuous stressor having been auditory might have interacted with divided attention to create additional stress.

In exploratory analyses, the effect of stress on activity-based PM was found in those that had military stress training and those that were not experienced in first-person shooter games. One explanation for those that had military stress training is that they may have acquired skills to focus under demanding conditions. Future research should investigate this possibility further.

Finally, the sample size achieved was not the sample size indicated from the power analysis. While the power analysis was conducted on the overall design, a sensitivity analysis was conducted on the main result, the effect of stress on activity-based PM. The effect size resulting from this analysis was ${\eta }_{p}^{2}=.097$, and according to the sensitivity analysis, an effect size of ${\eta }_{p}^{2}=.080$ is needed with a sample size of 101. However, the lack of the desired sample size still remains a limitation, and this effect should be replicated with a larger sample size.

## Conclusions

In the current study, we employed an ecologically valid experimental paradigm to explore the effects of stress and divided attention on PM tasks relevant to military contexts, focusing on *event-* and *activity-*based PM tasks. We believe that it is the first to assess the effect of stress on *activity*-based PM, and resulted in increased performance in the higher than lower stress group under a moderate level of stress, while providing additional support for no effects on *event*-based PM under a moderate level of stress.

Future research should focus on whether stress affects more demanding *event*-based PM tasks in the same way it can affect *time*- and *activity*-based tasks. Because high stress is innate in high-risk contexts, where PM failures can have a devastating effect, the relationship between stress and *activity*-, *event-*, and *time*-based PM necessitates further research under stress and attentionally demanding conditions to test the limits of this effect, and under what conditions performance may start to decline.

## Data Availability

The datasets generated during the current study are property of the government, but can be made available through formal processes through the Naval Postgraduate School.

## Abbreviations

HRV: Heartrate variability
PM: Prospective memory
RR: Inter-beat interval
STAI: State-trait anxiety inventory–state version
TSST: Trier social stress test

## Footnotes

1 ID badges were made using faces from Meissner and colleagues face database (http://iilab.utep.edu/stimuli.htm). Names were randomly put together using (a) last names from the 2010 US Census, and (b) first names from a list of male names from a 1980–1989 social security sample.

2 Statistics reported here slightly differ than those reported in the dissertation due to a previous error in reporting that did not account for removal of outliers.
